# The P450-Monooxygenase Activity and CYP6D1 Expression in the Chlorfenapyr-Resistant Strain of *Musca domestica* L.

**DOI:** 10.3390/insects15060461

**Published:** 2024-06-20

**Authors:** Kseniya Krestonoshina, Anastasia Melnichuk, Anna Kinareikina, Kseniya Maslakova, Liana Yangirova, Elena Silivanova

**Affiliations:** All-Russian Scientific Research Institute of Veterinary Entomology and Arachnology—Branch of Federal State Institution Federal Research Centre Tyumen Scientific Centre of Siberian Branch of the Russian Academy of Sciences (ASRIVEA)—Branch of Tyumen Scientific Centre SB RAS Institutskaya St. 2, Tyumen 625041, Russia; krutko.k.s@hotmail.com (K.K.); melnichukad1999@gmail.com (A.M.); kinareickina@yandex.ru (A.K.); k.y.maslakova@gmail.com (K.M.); lianayangirova137@gmail.com (L.Y.)

**Keywords:** chlorfenapyr, cytochrome P450 monooxygenases, expression level, insecticide resistance, mutation, *Musca domestica*

## Abstract

**Simple Summary:**

Enzymes of the detoxification system are known to play a crucial role in insecticide resistance in insects. The role of P450 monooxygenases in providing resistance to chlorfenapyr is ambiguous and of interest due to possible negative cross-resistance to pyrethroids and other insecticides, the resistance to which is ensured by increased activity of these enzymes. This study aimed to perform a biochemical and molecular evaluation of P450 monooxygenases in susceptible and chlorfenapyr-resistant strains of *Musca domestica* L. depending on sex. The chlorfenapyr-resistant strain of *M. domestica* L. did not differ from the susceptible strain in terms of the pattern of overall P450-monooxygenase activity: the larvae of both strains had lower enzymatic activity than the adults. However, the development of resistance to chlorfenapyr in house flies was accompanied by an increase in P450-monooxygenase activity without changes in CYP6D1 expression. PCR-RFLP analysis revealed a previously undescribed mutation in the promoter region of the CYP6D1 gene of *M. domestica*, the effect of which on the gene expression level was not detected.

**Abstract:**

The house fly *Musca domestica* L. is one of the most common insects of veterinary and medical importance worldwide; its ability to develop resistance to a large number of insecticides is well known. Many studies support the involvement of cytochrome P-450-dependent monooxygenases (P450) in the development of resistance to pyrethroids, neonicotinoids, carbamates, and organophosphates among insects. In this paper, the monooxygenase activity and expression level of CYP6D1 were studied for the first time in a chlorfenapyr-resistant strain of house fly. Our studies demonstrated that P450 activity in adults of the susceptible strain (Lab TY) and chlorfenapyr-resistant strain (ChlA) was 1.56–4.05-fold higher than that in larvae. In females of the Lab TY and ChlA strains, this activity was 1.53- and 1.57-fold higher, respectively (*p* < 0.05), than that in males, and in contrast, the expression level of CYP6D1 was 21- and 8-fold lower, respectively. The monooxygenase activity did not vary between larvae of the susceptible strain Lab TY and the chlorfenapyr-resistant strain ChlA. Activity in females and males of the ChlA strain exceeded that in the Lab TY strain specimens by 1.54 (*p* = 0.08) and 1.83 (*p* < 0.05) times, respectively, with the same level of CYP6D1 expression. PCR-RFLP analysis revealed a previously undescribed mutation in the promoter region of the CYP6D1 gene in adults of the Lab TY and ChlA strains, and it did not affect the gene expression level. The obtained results show that the development of resistance to chlorfenapyr in *M. domestica* is accompanied by an increase in P450-monooxygenase activity without changes in CYP6D1 expression.

## 1. Introduction

Insect pests cause substantial damage to agriculture, resulting in high financial and environmental costs worldwide. Despite the multitude of possible alternative pest control methods, pesticide application is the most commonly used method against insects of agricultural, veterinary, and medical importance [1,2]. As a consequence, long-term use of insecticides acts as a factor in the selection of insecticide-resistant insect populations [3,4]. Nowadays, more than 600 insect and mite species are resistant to at least one insecticide [5]. Resistance to 62 different insecticidal active ingredients has been documented in the house fly (*Musca domestica* L). [6]. Resistance to various pyrethroid and/or organophosphorus insecticides has been detected in field populations of Aedes, Culex, and *Anopheles genera* of mosquitoes worldwide [7,8,9]. The control of the brown planthopper *Nilaparvata lugens* caused the development of resistance to most of the currently used insecticides (neonicotinoids, pyrethroids, organophosphates, carbamates, pyridines) [10]. This issue is exacerbated by the cross-resistance phenomenon and the emergence of multi-drug-resistant insect populations [11,12]. For instance, populations of the Anopheles, Culex, Aedes, and Culiseta mosquito genera with multiple resistance to four groups of insecticides (COCs, OPCs, pyrethroids, carbamates) were documented in Senegal in 2014 and in Iran in 2000–2020 [13,14].

Enzymes of the detoxification system are known to play a crucial role in insecticide resistance in insects [15]. Cytochrome-P450-dependent monooxygenases (P450), encoded by the CYP gene family, are classified as phase 1 detoxification enzymes for many chemicals [16], like plant secondary metabolites (alkaloids, terpenoids, steroids, phenolics, and others, for example, furanocoumarins), and insecticides [17,18]. P450s have been proven to be involved in the development of resistance to insecticides such as pyrethroids [19], neonicotinoids, carbamates, and organophosphates [15,20]. For example, cytochrome P450 is associated with the resistance of the brown planthopper (*N. lugens*) to imidacloprid, dinotefuran, thiamethoxam, clothianidin, sulfoxaflor, and buprofezin [21]; of the cotton aphid (*Aphis gossypii*) to acetamiprid [22]; and of the southern house mosquito (*Culex quinquefasciatus*) to permethrin [23]. The mechanisms of P450-mediated insecticide resistance are associated with their broad substrate specificity [15,24], high catalytic activity [3], and an increase in some P450s isoforms (duplication, gene amplification, and changes in expression level) [4,25]. Many insect orders, including Coleoptera, Diptera, Hemiptera, Hemiptera, Hymenoptera, and Lepidoptera, have exhibited overexpression of P450 leading to increased resistance to pesticides [3]. To date, a great number of insect P450s involved in insecticide resistance through overexpression [23,26]/upregulation [27] have been identified and functionally analyzed. Based on phylogenetic studies, insect CYP gene families have been organized into four major clades: the Mito CYP mitochondrial clade and the clades CYP2, CYP3, and CYP4 [3]. In insects, most single-copy genes belong to the CYP2 and Mito CYP clades; most multicopy paralogs belong to the CYP3 and CYP4 clades [25]. Increased expression levels during insecticide resistance development have been observed for genes belonging to the following families: CYP4 [20], CYP6 [2], CYP9 [26], and CYP12 [20].

Chlorfenapyr (4-bromine-2-(4-chlorophenyl)-1-(ethoxymethyl)-5-(trifluoromethyl)-1-H-pyrrol-3-carbonitrile) is a pyrrole proinsecticide [28]. According to the Insecticide Resistance Action Committee (IRAC), chlorfenapyr is categorized into group 13, “Uncouplers of oxidative phosphorylation via disruption of the proton gradient” [5]. Oxidative removal of the N-ethoxymethyl group of chlorfenapyr by cytochrome P450 monooxygenases leads to the formation of a toxic form of the molecule identified as CL 303268, tralopyryl (4-bromine-2-(p-chlorophenyl)-5-(trifluoromethyl)-1H-pyrrole-3-carbonitrile), which disrupts oxidative phosphorylation in mitochondria and leads to disruptions of ATP synthesis, energy starvation of cells, and ultimately death [28,29]. Currently, chlorfenapyr is widely used as a non-repellent insecticide in the Americas, Europe, Africa, the Pacific, and the Middle East [30,31]. After evaluating the sensitivity of field populations of the cotton bollworm *Helicoverpa armigera* (Lepidoptera: Noctuidae) to 11 insecticides, researchers concluded that chlorfenapyr can be effective for the population control of this pest species [32]. In countries where chlorfenapyr-based formulations have been used for plant protection for a long time, the emergence of resistant pest populations has been reported [11,33,34,35]. For example, when assessing the sensitivity level of field populations of western flower thrips (*Frankliniella occidentalis* P. (Thysanoptera: Thripidae)) in China to commonly used insecticides, only three out of the fourteen populations studied had high sensitivity to chlorfenapyr, while the rest showed decreased sensitivity to this insecticide [35].

The mechanism of the development of resistance to chlorfenapyr has not been fully explored. It is known that resistance to chlorfenapyr can be produced by increasing the activity of the following enzymes: P450 monooxygenases and esterases in field populations of red spider mites (*Tetranychus urticae* Koch.) [36,37], glutathione S-transferases in the rosaceous leaf roller (*Choristoneura rosaceana* Har.) [38], and esterases in the cotton seed bug (*Oxycarenus hyalinipennis* Costa) [34]. A potential mechanism for the development of resistance to chlorfenapyr is also associated with a reduction in cuticle permeability [37]. On the other hand, some researchers suggest that enzymes of the detoxification system are not involved in the development of resistance to chlorfenapyr [39]. A recent study showed that exposure to sublethal concentrations of chlorfenapyr resulted in an increase in the expression level of one GST gene, several esterase genes, and one CYP gene (BmCYP4C) and a decrease in the expression of two other CYP genes (BmCYP340A3 and BmCYP339A1) in the domestic silk moth (*Bombyx mori*) [40]. Another study employing a transcriptomic analysis of the chlorfenapyr-resistant strain of the red spider mite (*T. urticae*) demonstrated a decrease in the expression levels of genes encoding detoxification systems (UGTs, GSTs, P450s, ABC-transporters) [41]. It was also recently found that only certain P450 groups effectively metabolize chlorfenapyr in *A. gambiae* (CYP6P3, CYP9J5, CYP9K), *A. aegypti* (CYP9J32), and *T. urticae* (CYP392D8) [41,42]. These studies demonstrate that the development of resistance to chlorfenapyr may be species-specific. Overall, the contribution of monooxygenases to resistance to chlorfenapyr is ambiguous. In a recent study on malaria vectors, it was demonstrated that pyrethroid-resistant specimens were more sensitive to chlorfenapyr [43]; thus, monooxygenases are of interest due to possible negative cross-resistance to pyrethroids and other insecticides, the resistance to which is associated with a magnification of these enzymes’ activity.

The house fly (*Musca domestica* L. (Diptera:Muscidae)) is a highly mobile cosmopolitan species with significant sanitary importance in medicine and the veterinary field [44,45,46,47,48] due to its ability to transmit human and animal diseases and pathogens [49,50,51,52], including bacterial dysentery, cholera, shigellosis, avian influenza, salmonellosis [45], staphylococcus, enterococcus, escherichia coli, typhoid fever, tuberculosis, ophthalmia, anthrax, and others [53]. It is possible for house flies to spread antibiotic-resistant bacteria [51]. This fly species is capable of rapidly developing resistance to applied insecticides [49]. To the best of the authors’ knowledge, to date, no field populations of *M. domestica* resistant to chlorfenapyr have been reported.

There are 146 P450 genes in the house fly genome [4], and CYP6D1 is among the best-characterized P450 genes involved in insecticide resistance in houseflies [48,54]. In *M. domestica*, CYP6D1 is mapped on autosome 1 [55]. CYP6D1 in *M. domestica* is responsible for the detoxification of pyrethroids with increased transcription of this gene and protein expression [56,57,58]. The involvement of CYP6D1 in *M. domestica* in the detoxification of neonicotinoids and spinosyns has also been reported [58,59]. The neonicotinoid-resistant strain 766b and spinosad-resistant strain 791spin had higher expression levels of CYP6D1 (along with the other 19 of the 100 P450 genes) than specimens of the WHO-SRS reference susceptible strain [59]. The multi-resistant strain (with resistance to pyrethroids, dimethoate, propetamphos, methomyl and azamethiphos, cyromazine, and fipronil) also showed overexpression of CYP6D1, along with CYP6A1 and CYP6D3 [58]. In the multi-resistant ALHF strain, 11 genes with >2-fold increased expression compared to susceptible aabys and CS strains were identified. The isolated genes were predominantly related to CYP4 and CYP6 (CYP4G13, CYP4G99, CYP4S24, CYP4E10, CYP4E11, CYP6A36, CYP6A40, CYP6A52, CYP6A58, CYP6D3, CYP6D10) [60].

The above examples demonstrate that studies on the involvement mechanisms of the P450 monooxygenase system in resistance development in house flies are generally carried out on specimens from field populations or those resistant to pyrethroids, neonicotinoids, and OPCs. So far, similar studies utilizing a chlorfenapyr-resistant strain of house flies have not been conducted. Consequently, this study aimed to perform an evaluation of P450 monooxygenase activities and CYP6D1 expression in susceptible and chlorfenapyr-resistant strains of *M. domestica*. The gene expression profiling of CYP6D1 in females and males of insects was performed; this will contribute to the understanding of the resistance formation mechanisms in specimens of different sexes.

## 2. Materials and Methods

### 2.1. Insects

The objects of this study were larvae and 3–5-day-old adults of two laboratory strains of the house fly (*Musca domestica* L.), namely, Lab TY and Lab UF, that were barred from contact with insecticides, as well as specimens of the chlorfenapyr-resistant strain (ChlA). The Lab TY strain was obtained from Novosibirsk State Agricultural University in 2009, while the Lab UF strain was obtained from the Institute of Biochemistry and Genetics UFRC RAS in 2023. The chlorfenapyr-resistant strain was previously selected with chlorfenapyr based on specimens of the Lab TY strain in the laboratory of veterinary problems in animal husbandry of the ASRIVEA Branch of Tyumen Scientific Centre SB RAS, with financial support from the Russian Foundation for Basic Research (RFBR) within the framework of scientific project No. 19-016-00059 [61].

### 2.2. Assay of P450-Activities

Homogenates were prepared from each specimen of *M. domestica* at low temperatures with the addition of 0.1 M of phosphate buffer (pH 7.6), containing 1 mM of ethylenediaminetetraacetic acid (EDTA), 1 mM of N-phenylthiourea (PTU), 1 mM of phenylmethylsulfonyl fluoride (PMSF), 1 mM of 1,4-dithioerythritol (DTE), and 20% Triton X-100. The supernatant before centrifugation was used to determine the monooxygenase activities; the supernatant obtained after centrifugation (2 min, 12,500 rpm) was used to determine protein concentrations via the Lowry protein assay, using bovine serum albumin solutions to construct a calibration curve. The determination of enzyme activity was performed on 96-well microplates (MiniMed, Suponevo, Russia) using a Multiskan FC microplate photometer (Thermo Fisher Scientific Inc., Waltham, MA, USA).

The functional activity of the cytochrome P450 monooxygenases was assessed according to the total content of heme at 620 nm in end-point mode [62]. The reaction mixture contained 20 µL of homogenate, 60 µL of 90 mM potassium phosphate buffer (pH 7.2), 200 µL of a working solution of 0.2% TMBZ with 250 mM of sodium acetate buffer (pH 5.0), and 25 µL of 3% hydrogen peroxide. Cytochrome C solutions were used to construct a calibration curve. P450 monooxygenase activity was represented as the µg of cytochrome C/mg of protein.

### 2.3. Determination of CYP6D1 Expression Level via Real Time PCR

Seven-day-old puparia and 3–5-day-old adults of the Lab TY and Lab UF *M. domestica* strains were used to assess reference genes before studying the expression levels of CYP6D1. Total RNA (tRNA) was extracted from each specimen using high-purity totalRNA kit microcolumns (Magen, Guangzhou, China) and treated with DNase I. The quantity and quality of tRNA were assessed using a Nano-500 spectrophotometer (Allsheng, Hangzhou, China) according to the ratio of optical density at a wavelength of 260/280 nm and by subjecting an aliquot to 1% agarose gel electrophoresis. The first-strand cDNA was synthesized using the MMLV RT kit (Evrogen, Moscow, Russia) according to the manufacturer’s instructions. Primers were designed using Primer3 (version 2.6.1) software in combination with Beacon designer 5.0. The specificity of primers for RT-qPCR was tested through the alignment of the primer sequence in NCBI BLAST, analysis of melting curves of the PCR product (Figure S1), and electrophoresis of PCR products in 6% PAAG or 2% agarose gel (Figures S2 and S3).

Real-time PCR was carried out using BioMaster HS-qPCR SYBR Blue mixture (Biolabmix, Novosibirsk, Russia) on iQ5 cyclers (Bio-Rad, Hercules, USA) and Gentier 96E (Tianlong, Xi’an, China). Each reaction was performed at least in triplicate, and a non-template control and a negative control were used to exclude contamination of the reagents. A standard curve was generated for each selected *M. domestica* gene using 10-fold serial dilutions of the pooled cDNA. The PCR reactions were performed according to the same protocol for each primer as follows: initial preincubation at 95 °C for 5 min, followed by 35 cycles of denaturation at 95 °C for 1 min, annealing at 59 °C for 20 s, and elongation at 72 °C for 20 s.

Statistical analysis of the data and their ranking by normalization coefficient were carried out using the RefFinder program based on raw cycle threshold (Ct) values. Ct values were calculated using standard cycler software at a threshold line value of 50.

*M. domestica* adults (10 females and 10 males from each strain) were used to study CYP6D1 gene expression. Primers (F 5′-AGAACGCTTTGCCGATGAG-3′; R 5′-GCTACCTTGGAATTGATAACGC-3′) were designed using Beacon designer software 5.0. The amplification protocol used was as follows: preincubation at 95 °C for 5 min, followed by 35 cycles of denaturation at 95 °C for 45 s, annealing at 58.4 °C for 15 s, and elongation at 72 °C for 20 s.

### 2.4. DNA Preparation and PCR-RFLP Analysis of CYP6D1

DNA for PCR-RFLP analysis was extracted from each specimen using the alkaline lysis method [63]. The primer pair (S35, AGCTGACGAAATTGATCAATCAGT, and AS2, CATTGGATCATTTTTCTCATC) was designed based on sequence No. AF200191.1 from the NCBI database. Primers S35/AS2 synthesize a 732 bp fragment for the pyrethroid-resistant genotype (with 15 bp-insert mutation and a 711 bp fragment for the susceptible one (without 15 bp-insert mutation) [64]. The reaction medium for PCR included 1.5 µL of total DNA, 4 µL of ready-to-use for PCR 5X ScreenMix-HS (Evrogen, Moscow, Russia), 0.4 µL of 25 µM of each primer, and 13.7 µL of purified sterile water (18.2 µS/cm). The amplification process was performed using a GeneExplorer GE-96G device (Bioer, Hangzhou, China) under the following temperature conditions: 94 °C for 5 min, followed by 94 °C for 20 s, 61 °C for 30 s, 72 °C for 30 s (5 cycles), 94 °C for 20 s, 59 °C for 30 s, 72 °C for 30 s (35 cycles), and 72 °C for 10 min. The PCR product was used for further restriction enzyme analysis using the enzyme Hpy188III (New England Biolabs Inc., Ipswich, MA, USA) that is able to cut only the PCR product without a 15 bp-insert mutation. The presence of the 732 bp band indicates a genotype with a mutation, and the presence of two bands (432 bp and 279 bp) indicates a genotype without mutations. The presence of 732 bp, 432 bp, and 279 bp fragments is characteristic of a heterozygote [64]. A fragment of the *M. domestica* acetylcholinesterase gene (609 bp) was used as a positive control. Hpy 188III cuts this fragment into 370 bp, 140 bp, and 70 bp. Reaction conditions for restriction were 1 h at 37 °C and 20 min at 60 °C. PCR-RFLP results were visualized on a 2% agarose gel stained with ethidium bromide.

### 2.5. Data Analysis

The statistical analysis of the enzyme activity results and the expression level results was performed using a one-way ANOVA test, Dunn’s test for multiple comparisons, and Mann–Whitney test for comparing females and males using the Python programming language with a library for statistical analysis (NumPy and SciPy packages). A significance level of *p* < 0.05 was used to indicate when the identified differences were statistically significant.

## 3. Results

### 3.1. Cytochrome P450 Monooxygenase Activity

**Figure 1:**
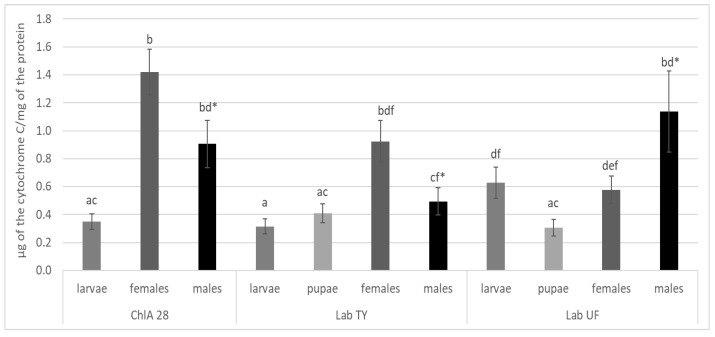
The cytochrome P450 monooxygenase activities in *M. domestica* of chlorfenapyr-susceptible (Lab UF, Lab TY) and -resistant (ChlA, generation 28) strains. The values with the same letters are not significantly different according to Dunn’ test comparisons at *p* < 0.05. An asterisk indicates a statistically significant difference between males and females from the same strain according to Mann–Whitney test at *p* < 0.05.

The total monooxygenase activity results are shown in Figure 1. The Lab TY strain and the ChlA strain derived from it had lower P450 activity at preimaginal developmental stages (larvae, pupae) than adult females and males by a factor of 1.56–4.05. In the Lab UF strain, the activity in males alone exceeded that of the larvae and pupae by 1.81 and 3.74 times, respectively. All three strains showed sexual dimorphism in P450 activity: the activity in females of the Lab TY and ChlA strains was 1.53- and 1.57-fold higher (*p* < 0.05), respectively, and in contrast, the activity in females of the Lab UF strain was 1.97-fold lower (*p* < 0.05) than that in males. According to the results of Dunn’s test, differences in activity between the specimens of the two laboratory strains were detected. The activity levels in larvae and males of the Lab UF strain were 1.98 and 2.30 times higher (*p* < 0.05), respectively, than those in the specimens of the Lab TY strain. The activity in females and males of the ChlA strain was higher than that of specimens of the Lab TY strain by factors of 1.54 (*p* = 0.08) and 1.83 (*p* < 0.05), respectively. In larvae of the Lab TY and ChlA strains, the activity did not differ.

### 3.2. Analysis of CYP6D1 Gene Expression

The candidates for the reference genes were selected based on the following two criteria: (1) highest frequency of use in similar studies and (2) functional class difference. The following genes were selected: 18S rRNA, 18S (GeneBank: 1135100218), a cytosolic small ribosomal subunit; GAPDH, (GeneBank: DQ386609.1), a glyceraldehyde 3-phosphate dehydrogenase, which is an oxidoreductase in glycolysis and gluconeogenesis; ribosomal protein S18, RPS18 (GeneBank: KC424479.1), a component of the 40S subunit of the ribosome and elongation factor 1; and EF-1 (GeneBank: GQ465788.1), which catalyzes the GTP-dependent binding of aminoacyl-tRNA and ribosome (Table S1).

Ct values representing the expression level of the reference genes selected for the study ranged from 14 to 27 cycles. RPS18 was characterized by the lowest Ct threshold cycle values (from 14,855), indicating the highest level of transcripts. Expression of the 18S gene was characterized by late threshold cycles (>25 Ct) and thus low expression levels, which do not meet the criteria for reference genes. Ranking by normalization factor was performed in the RefFinder program, which uses multiple algorithms to evaluate reference genes: Delta Ct, BestKeeper, NormFinder, and Genorm. Based on the ratings of each program, an individual gene is assigned a corresponding weight value, and the geometric mean of their weights is calculated for the overall final rating. The results of all four algorithms differed slightly from each other; the final scores of all algorithms in the RefFinder program were ranked as follows: EF-1 (M = 1) > RPS18 (M = 1682) > 18S (M = 3) > GAPDH (M = 4), where EF-1 is the most stable gene for all three studied *M. domestica* populations, while GAPDH is the least stable gene.

**Figure 2:**
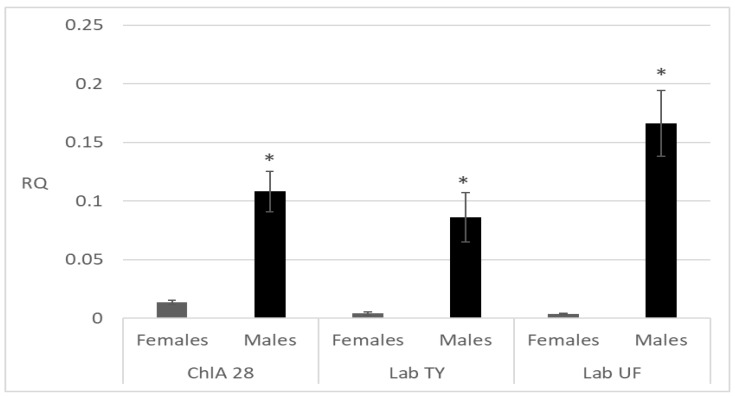
The relative CYP6D1 expression in *M. domestica* of chlorfenapyr-susceptible (Lab UF, Lab TY) and -resistant (ChlA, generation 28) strains. RQ ± SD (Relative Quantitation ± Standard deviation) was assessed as the difference between the cycle threshold (Ct) values of the reference and CYP6D1 genes. Asterisks indicate statistically significant differences between males and females from the same strain according to Mann–Whitney test at *p* < 0.05.

The gene expression profiling of CYP6D1 in adults did not reveal statistically significant differences between the strains studied. All strains showed sex-dependent differences in gene expression. The gene expression profiling according to the Mann–Whitney U test demonstrated 8-, 21-, and 45-fold increased expression in males of the ChlA, Lab TY, and Lab UF strains, respectively, compared to the expression in females (Figure 2).

### 3.3. CYP6D1 Genotyping

**Figure 3:**
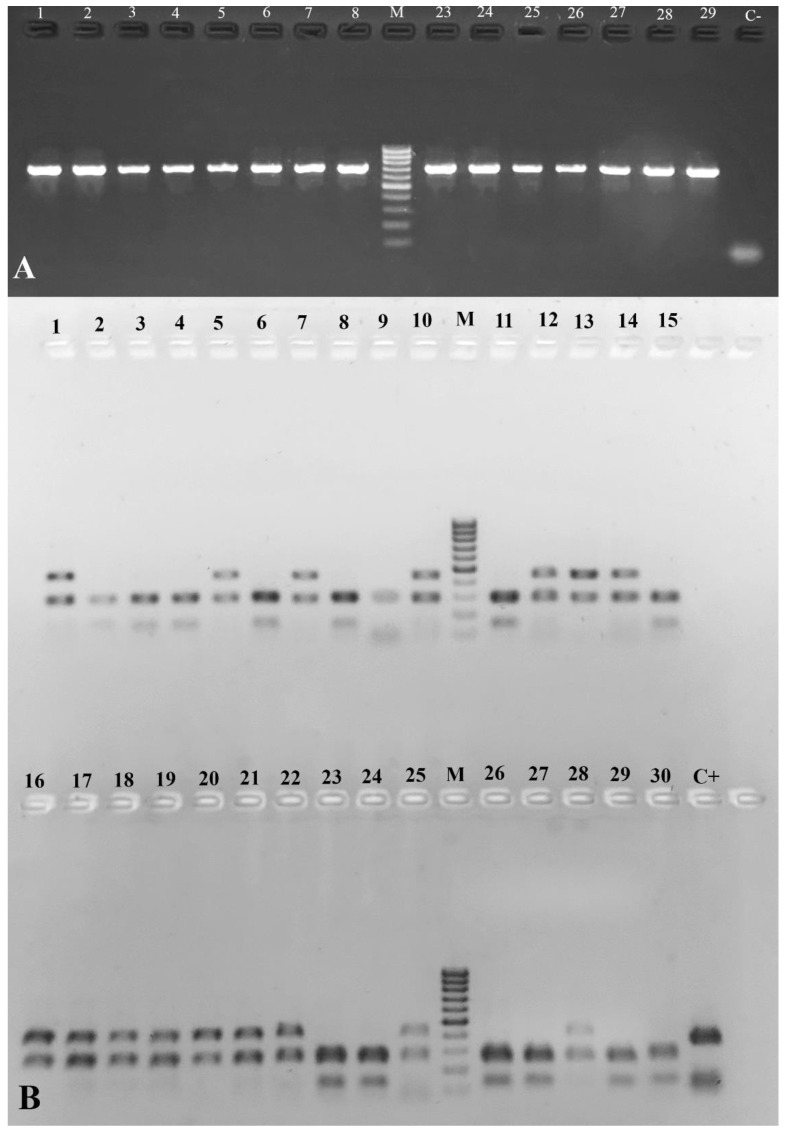
Electropherogram of PCR products obtained via DNA amplification with primers S35/AS2 (**A**) and restriction products obtained via cleavage with Hpy188III (**B**). M—molecular weight marker of 100 bp DNA fragments; 1–10—females from the ChlA strain; 11–20—females from the Lab UF; 21–30—females from the Lab TY; C+—positive control (*M. domestica ace*, 609 bp); C−—negative control.

**Figure 4:**
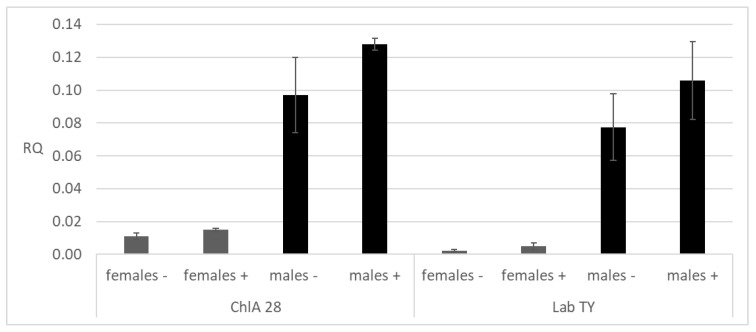
The relative CYP6D1 expression in chlorfenapyr-susceptible (Lab TY) and -resistant (ChlA, generation 28) strains of *M. domestica*. RQ ± SD (Relative Quantitation ± Standard deviation) was assessed as the difference between the cycle threshold (Ct) values of the reference and the CYP6D1 genes; −/+ indicates absence/presence of the new suggested mutation in the CYP6D1 promoter region.

A total of 60 samples of 10 females and 10 males from three different strains of *M. domestica* (ChlA, Lab TY, Lab UF) were analyzed. The sought mutation of 15 bp was not detected in any of the tested samples. However, a new mutation, not previously described, was identified in the females and males of *M. domestica* (Figure 3 and Figure S4). The electropherogram shows that in some specimens, the 432 bp band is additionally cut into two fragments (approx. 280 bp and 150 bp) by the Hpy188III enzyme. Based on the results of the Mann–Whitney U test, no statistical differences were found in the expression levels between specimens with and without the mutation (Figure 4).

## 4. Discussion

Although chlorfenapyr has been used as an insecticide and acaricide for quite a long time already [28,30] and chlorfenapyr-resistant populations of insects and ticks are known to exist [11,33,34,35], the mechanism of developing resistance to it has not been fully investigated. Cytochrome-P450-dependent monooxygenases (P450) have an important function in insect defense against insecticides; at the same time, through P450-monooxygenase activation, chlorfenapyr is metabolized into the toxic compound tralopyril [28,29]. In this regard, the role of monooxygenases in chlorfenapyr resistance is both interesting and complex. A presumed protective reaction of insects in response to prolonged exposure to chlorfenapyr could be qualitative and quantitative changes in the P450-monooxygenase system.

According to the obtained results, the P450 activity profile of the Lab TY strain *M. domestica* and the chlorfenapyr-resistant ChlA strain derived from it matched: the activity in larvae was lower than that in adults, and that in males was lower than that in females. In our opinion, this is important because it shows that in preimaginal stages, these insects may be more defenseless against insecticides with respect to detoxification. The observed increased P450 activity in females and males of the ChlA strain relative to the activity of specimens of the Lab TY strain suggests that P450 is associated with chlorfenapyr resistance, especially in males, but not in the larvae. Previously, during the selection process for resistance to chlorfenapyr, a statistically significant increase in the specific activity of P450 in the sixth generation of ChlA strain house flies of 1.7 times for females and 2.1 times for males, as well as a 1.4-fold increase in the eighth generation in females, relative to the values of the Lab TY control strain was revealed [65].

As a rule, the development of resistance to insecticides is associated with the activation of the monooxygenase system [17]. For instance, the study by Markussen and Kristensen [58] demonstrated that P450 monooxygenases contribute significantly to the formation of neonicotinoid resistance in house flies of lab insecticide-selected strains and field populations. This is supported by studies by other authors: the P450 monooxygenase activity in females of *M. domestica* was 1.93 times higher in a thiamethoxam-resistant strain [66] and 4.3 times higher in a imidacloprid-resistant strain [67] compared to the enzyme activity of the control group. An increase in P450 monooxygenase activity was also detected in females (10.7-fold) and males (2.3-fold) of the spinosad-resistant strain of *M. domestica* compared to susceptible specimens [68].

In addition, our study revealed sexual dimorphism in P450 activity: males of the chlorfenapyr-susceptible Lab UF strain had higher activity, and males of the chlorfenapyr-resistant ChlA strain had lower activity than females of the corresponding strain. Similar results were obtained by Zhang [68] in a sex-dependent study on spinosad resistance in *M. domestica*. According to the results of a Mann–Whitney U test, sexual dimorphism in the expression level of CYP6D1 was detected, which was most pronounced in the Lab UF strain specimens. The results we obtained on the level of CYP6D1 expression in *M. domestica* adults on the basis of sex are comparable with the results obtained by other authors. For instance, the expression level of CYP6D1 was higher in males than in females of a susceptible strain (WHO-SRS), a multi-resistant strain (791a), and neonicotinoid-resistant strains (766b, 791imi, 791tmx) [58] but not in a spinosad-resistant strain [69]. Previously, different expression patterns of P450 genes in females and males of *M. domestica* were also described for CYP4G2 and CYP6A5v2 [68]. Notably, despite higher CYP6D1 expression levels in males of the Lab TY and ChlA strains, overall P450-monooxygenase activity was higher in females.

Given chlorfenapyr’s mode of action, the response to the insecticidal challenge was expected to be a decrease in P450-monooxygenase activity in specimens of the chlorfenapyr-resistant strain, but we observed the opposite. Usually, in cases of P450-mediated insecticide resistance, CYP expression levels in insects appear to be elevated [2,20,26]. For the house fly, this has been demonstrated in pyrethroid-, neonicotinoid-, and spinosad-resistant strains [59], as well as in specimens with multiple resistance (resistance to pyrethroids, dimethoate, propetamphos, methomyl and azamethiphos, cyromazine, and fipronil) [58]. At the same time, in the multi-resistant ALHF strain of *M. domestica*, increased expression (more than 2-fold) was typical mainly for the genes of the CYP4 and CYP6 groups [60]. Only recently has discussion started on the downregulation of P450-monooxygenase genes involved in proinsecticide activation serving as an insecticide resistance mechanism [25,43]. A study on transcriptomic analysis of the chlorfenapyr-resistant strain of *T. urticae* showed that different groups of P450 genes demonstrated transcriptional differences (upregulation, downregulation, or no change) upon chlorfenapyr selection [43]. In our study, the expression levels of CYP6D1 did not differ between adults of the chlorfenapyr-susceptible Lab TY strain and the chlorfenapyr-resistant ChlA strain. Therefore, we suggest that CYP6D1 does not participate significantly in chlorfenapyr resistance in house flies, and the likelihood of developing of pyrethroid resistance in the ChlA strain is low. The increased P450 activity of the chlorfenapyr-resistant specimens might be caused by either other quantitative changes (the amplification or increased expression of other CYPs) or qualitative changes.

The obtained data on the expression level of CYP6D1 in adults of the chlorfenapyr-susceptible Lab TY and resistant ChlA strains are consistent with the results of PCR-RFLP analysis, according to which a 15-bp insertion in the 5’-flanking region of CYP6D1 was not detected in either the susceptible strains or chlorfenapyr-resistant strains. It is known that this insertion determines the future of the CYP6D1v allele responsible for increased CYP6D1 expression in pyrethroid-resistant *M. domestica* [64,70]. Unexpectedly, two additional fragments (approximately 280 bp and 150 bp) were detected during the restriction of CYP6D1 amplicons by the Hpy188II enzyme, indicating the presence of a previously undescribed mutation (Figure 3 and Figure S4). Analysis using the Mann–Whitney U test revealed no statistically significant differences in the expression levels between specimens with and without the mutation (Figure 4); it is most likely the case that this mutation does not affect the regulation of CYP6D1 gene expression level. Since the mutation we found occurred in both the chlorfenapyr-resistant ChlA strain and the susceptible Lab TY strain, it probably does not affect chlorfenapyr detoxification either.

**Figure 5:**
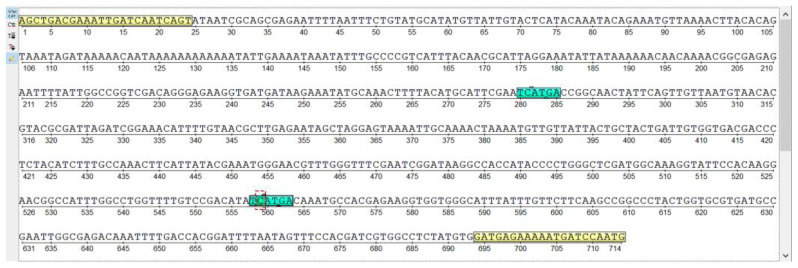
Analysis of new suggested mutation in CYP6D1 promoter region using Ugene 49.1. The supposed mutation site is based on sequence CYP6D1 № AF200191.1 from NCBI.

Further analysis of the promoter region of the CYP6D1 gene using the Ugene 49.1 program revealed a region similar to the recognition site of the Hpy188III restrictase. Thus, if a single cytosine nucleotide insertion occurs at position 11136 in the AF200191.1 sequence (NCBI), a site for restrictase will form (Figure 5).

## 5. Conclusions

In summary, the evaluation of P450 monooxygenases in susceptible and chlorfenapyr-resistant strains of *M. domestica* revealed sexual dimorphism in the monooxygenase activity and CYP6D1 expression levels in these strains of *M. domestica*. Presumably, CYP6D1 in males of *M. domestica* plays a more significant role in the P450-monooxygenase system than it does in females. The chlorfenapyr-resistant strain of *M. domestica* did not differ from the laboratory susceptible strain in the pattern of overall P450-monooxygenase activity: larvae of both strains had lower enzymatic activity than adults. However, we observed an increase in the P450 activity without changes in CYP6D1 expression level in both females and males of the chlorfenapyr-resistant strain compared to specimens of the susceptible strain. Consequently, we suggest that monooxygenases are involved in the development of resistance to chlorfenapyr in house flies, but it is not CYP6D1 that is activated. Therefore, further studies on the expression of particular CYPs in specimens of the chlorfenapyr-resistant strain are required. A new mutation was found in the promoter region of the CYP6D1 gene in the Lab TY and ChlA strains, which is a recognition site for the Hpy188III restriction enzyme. The obtained results will contribute to a better understanding of the defense system action against xenobiotics and the development of insecticide resistance in insects depending on sex.

In general, insect control practices require rotation of insecticides with different modes of action to prevent insecticide resistance. The use of insecticide mixtures is also thought to slow the development of resistance. For example, the WHO recommends using a pyrethroid–chlorfenapyr mixture for malaria vector control. To effectively counter chlorfenapyr resistance, it is necessary to comprehensively understand the mechanism of its development. Since we found an increase in P450 activity in adult chlorfenapyr-resistant house flies, subsequent studies may focus on studying the levels and regulation of expression of different CYP genes, the contribution of other components of the detoxification system (esterase, GST, UDP-transferase, ABC-transporters), and the role of the insect cuticle in generating resistance to chlorfenapyr.

## Data Availability

The data presented in this study are available on request from the corresponding author.

## Supplementary Materials

The following supporting information can be downloaded at https://www.mdpi.com/article/10.3390/insects15060461/s1, Figure S1: Analysis of the melting of RT-qPCR products using the Gentier 96E standard software amplifier (TianLong, China); Table S1: Genes evaluated in this study; Figure S2: Agarose gel (2%) electrophoresis of RT-qPCR products. M—molecular weight marker of DNA fragments (50 bp–700 bp) (Evrogen, Russian Federation); 1–4 fragments of CYP6D1 (114 bp); K-negative control based on RNA; Figure S3: Agarose gel (2%) electrophoresis of RT-qPCR products obtained with primers for reference genes. M—molecular weight marker of DNA fragments (50 bp-700 bp) (Evrogen, Russian Federation); 1-3-RPS18 (fragments of 149 bp); 4-6-EF-1 (fragments of 91 bp); Figure S4: Electropherogram of restriction products formed by cleavage with Hpy188III. M—molecular weight marker of DNA fragments 100 bp; 1–40—males from the ChlA strain; 41–50—males from the Lab UF; 51–60—males from the Lab TY; C+—positive control (*M. domestica ace*, 609 bp).
