# Supplementary material for: The P450-Monooxygenase Activity and CYP6D1 Expression in the Chlorfenapyr-Resistant Strain of Musca domestica L
Source: Insects. 2024 Jun 20;15(6):461. doi: 10.3390/insects15060461 (PMC11203901; doi:10.3390/insects15060461)
Supplement: Supplementary file 1 [file insects-15-00461-s001.zip › insects-3024849-supplementary.pdf]

Supplementary Materials

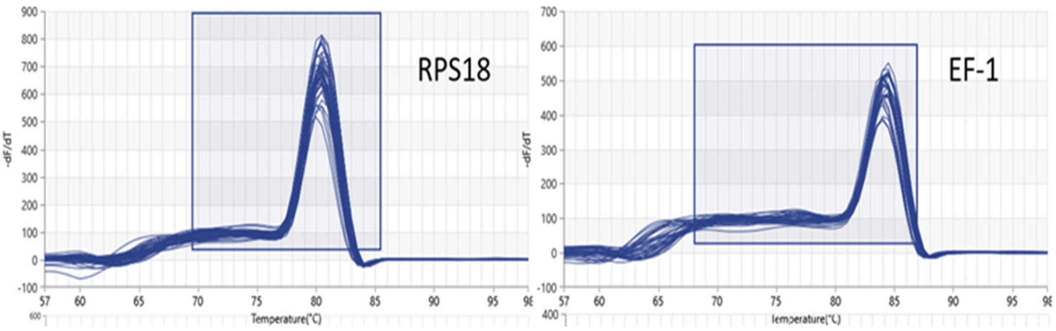

Figure S1. Analysis of the melting of RT- qPCR products using the Gentier 96E standard software amplifier (TianLong, China)

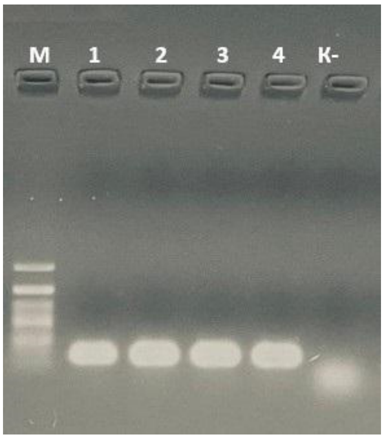

Figure S2. Agarose gel (2 %) electrophoresis of RT-qPCR products. M - molecular weight marker of DNA fragments (50 bp-700 bp) (Evrogen, Russian Federation); 1-4 fragments of CYP6D1 (114 bp); K- negative control based on RNA

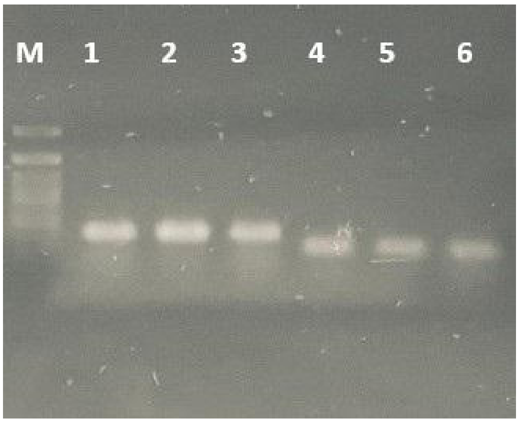

Figure S3. Agarose gel (2 %) electrophoresis of RT-qPCR products obtained with primers for reference genes. M - molecular weight marker of DNA fragments (50 bp-700 bp) (Evrogen, Russian Federation); 1-3 - RPS18 (fragments of 149 bp); 4-6 - EF-1 (fragments of 91 bp).

Table S1. Genes evaluated in this study

Gene	Name	Nucleotide sequence (5`→ 3`) of primers (forward/reverse)	Tm	Length of PCR product (bp)	GenBank ID	Method
RPS18	Рибосомальный белок s18	ATCGTCACCATCATCTCCAAC<br>TTCTTCAAGCGTTCCAAATCG	59	149 bp	KT006855.1	RT-qPCR
EF -1	Фактор элонгации	TAAGGAAGGTAACGCTGAAGG<br>CAAGGGCAAACGCAAAGG	59	91 bp	AF503149.1	RT-qPCR
CYP6D1	CYP6D1	AGAACGCTTTGCCGATGAGG<br>GCTACCTTGGAATTGATAACGC	58.4	114 bp	AF064795.1	RT-qPCR
CYP6D1	CYP6D1	AGCTGACGAAATTGATCAATCAGT<br>CATTGGATCATTTTTCTCATC	56.5	711 bp	№AF200191.1	PCR- RFLP

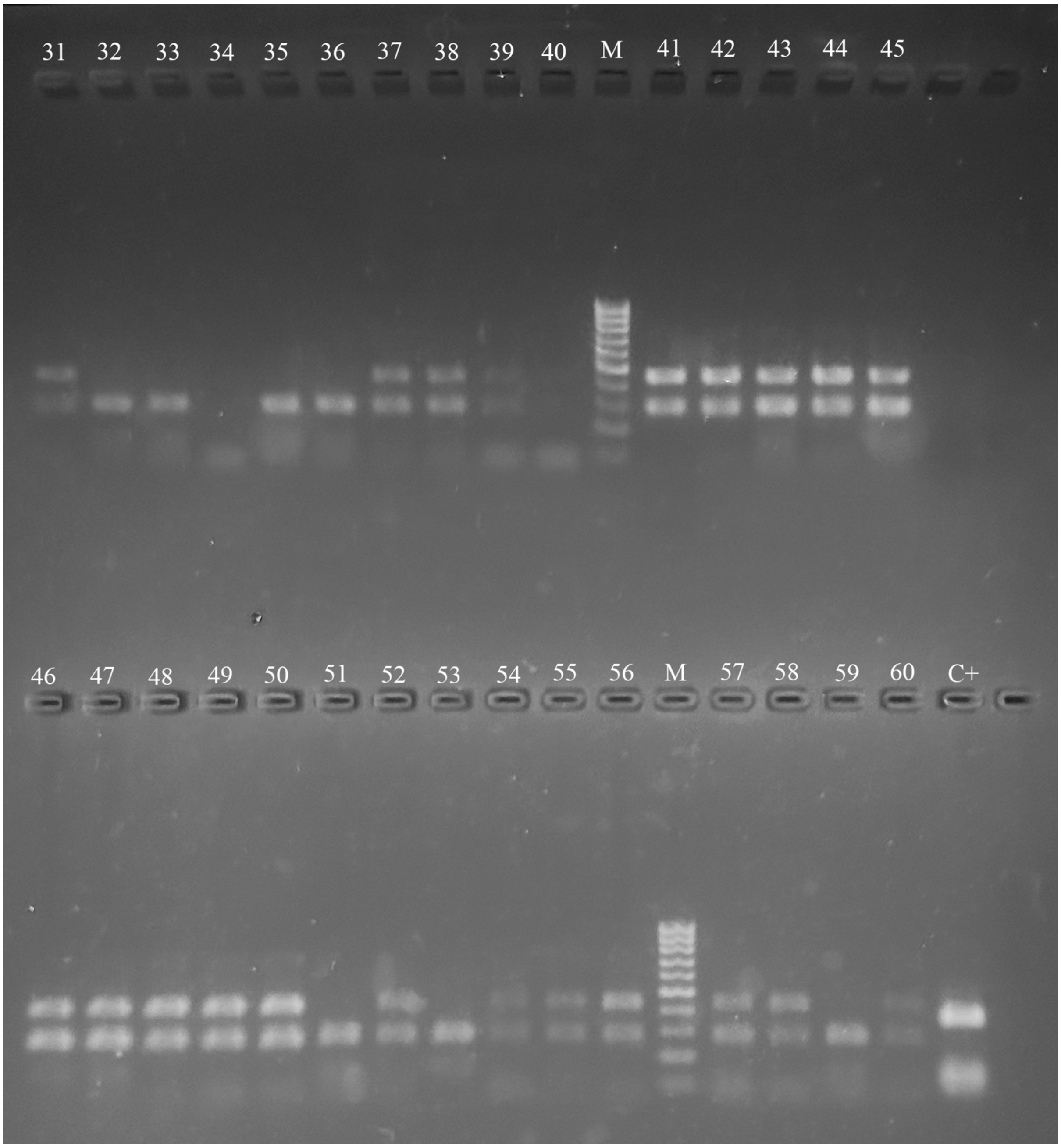

Figure S4. Electrophoregram of restriction products by cleavage with Hpy188III. M - molecular weight marker of DNA fragments 100 bp; 31-40 – males from the ChlA strain; 41-50 – males from the Lab UF; 51-60 – males from the Lab TY; C+ – positive control (*M. domestica ace*, 609 bp).
